# Microstructure and Mechanical Properties of Metal Foams Fabricated via Melt Foaming and Powder Metallurgy Technique: A Review

**DOI:** 10.3390/ma15155302

**Published:** 2022-08-01

**Authors:** Bisma Parveez, Nur Ayuni Jamal, Hazleen Anuar, Yusilawati Ahmad, Abdul Aabid, Muneer Baig

**Affiliations:** 1 Department of Manufacturing and Materials Engineering, Kulliyyah of Engineering, International Islamic University Malaysia, Kuala Lumpur 53100, Malaysia; mirbisma5555@gmail.com (B.P.); hazleen@iium.edu.my (H.A.); 2 Biotechnology Engineering Department, Kulliyyah of Engineering, International Islamic University Malaysia, Kuala Lumpur 53100, Malaysia; yusilawati_ahmadnor@iium.edu.my; 3 Department of Engineering Management, College of Engineering, Prince Sultan University, P.O. Box 66833, Riyadh 11586, Saudi Arabia; aabidhussain.ae@gmail.com (A.A.); mbaig@psu.edu.sa (M.B.)

**Keywords:** compressive properties, Gibson and Ashby model, mechanical properties, metal foams, melt foaming, powder metallurgy

## Abstract

Metal foams possess remarkable properties, such as lightweight, high compressive strength, lower specific weight, high stiffness, and high energy absorption. These properties make them highly desirable for many engineering applications, including lightweight materials, energy-absorption devices for aerospace and automotive industries, etc. For such potential applications, it is essential to understand the mechanical behaviour of these foams. Producing metal foams is a highly challenging task due to the coexistence of solid, liquid, and gaseous phases at different temperatures. Although numerous techniques are available for producing metal foams, fabricating foamed metal still suffers from imperfections and inconsistencies. Thus, a good understanding of various processing techniques and properties of the resulting foams is essential to improve the foam quality. This review discussed the types of metal foams available in the market and their properties, providing an overview of the production techniques involved and the contribution of metal foams to various applications. This review also discussed the challenges in foam fabrications and proposed several solutions to address these problems.

## 1. Introduction 

Metal foams are cellular structures comprising solid materials with a large portion of gas-filled pores by volume. Due to their cellular structure, metal foams possess a set of unique mechanical and physical properties. These properties allow them to become highly efficient in several engineering applications, notably in components for blast resistance, fire resistance, thermal insulation, foam core sandwich panels, and sound and vibration damping [1,2]. In addition, they are recyclable, with no disposal issues [3]. Consequently, these materials have attracted immense attention in recent years. One of the exceptional features of foams is that their mechanical properties are flexible, and their pore size, geometry, density, and choice of foaming material can be controlled. When used as energy absorption materials, these foams could go through substantial deformations under nearly constant stress [4]. With the rapid advancements in defence, aerospace, and automotives, there is an increasing demand for lightweight materials with high specific strength, better fuel efficiency, and high energy absorption capacity to withstand impact forces [5,6]. Thus, their good mechanical, acoustic, electrical, thermal, and chemical properties make them ideal for structural and functional applications [6,7]. Metal foams generally consist of aluminium (Al), nickel (Ni), magnesium (Mg), copper (Cu), zinc (Zn), and steel. In particular, Al and their alloys are widely used as non-flammable materials for thermal and sound insulation, sandwich cores, mechanical damping, lightweight panels and impact resistance in transportation, strain insulators, and vibration control [8,9,10]. 

These foams are mostly developed by the addition of foaming agents or space holders in the matrix metal. Several methods have been used for foam development. These methods include adding foaming agent in liquid melt [11], compaction of metal powder and blowing agent [12], blowing gas [13], etc. This review discusses the microstructure of various metal foams, their mechanical properties, manufacturing methods, and industrial applications. Evaluating the properties of metal foams developed through different techniques is essential to find the optimum manufacturing strategies and the effects of various parameters on their microstructure and strength. After introducing different types of metal foams, we discuss the microstructure and properties of the metal foams. The fabrication techniques such as melt foaming and powder metallurgy techniques are discussed in the next section, Section 6 and Section 7 discusses the industrial applications of metal foams and the way forward respectively. 

## 2. Types of Metal Foams

Foam structures are divisible into two types, i.e., the open- cell foams and closed-cell foams, as listed in Table 1. In open-cell foams, pores are connected to allow matters to pass through them. By contrast, pores are isolated in closed-cell foams. In general, open-cell foams are preferred for functional applications, such as in filters, catalyst supports, heat exchangers, etc., and closed-cell foams find applications in silencers, automobiles, bearings, sound and energy absorbers, etc. [14].

### 2.1. Open-Cell Foams

**Figure 1:**
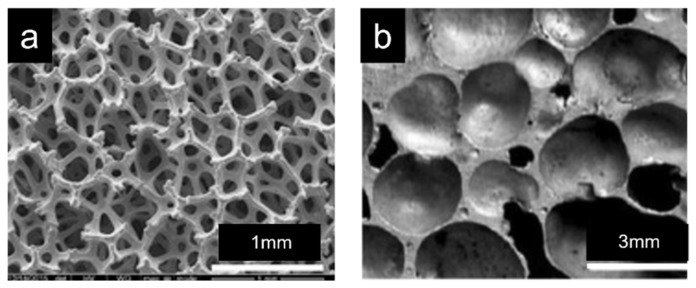
The optical micrograph of microstructure of (**a**) open-cell metal foam [15] and (**b**) closed-cell metal foam [28].

In open-cell foams, no film occurs between adjacent cells in the matrix material. A larger effective surface area (10–1000 times) is exposed to the surroundings compared to the dense material. They have sponge-like interconnected pores, as shown in Figure 1a [15]. Metal foams have a high specific surface area. These features make them suitable materials for heat exchangers, sound absorbers, catalysts, hydrogen storage, filter elements, etc. [16,17]. Meanwhile, high-porosity metal foams are commonly used in several real-world devices, including heat exchangers [18,19,20,21,22], fuel stacks [23], solar collectors [24,25], heat storage [26,27], etc. These foams possess excellent thermal properties, high permeability, high conductivity, and volume-to-area ratio. 

### 2.2. Closed-Cell Foams

Cells in the closed-cell foams are separated by a thin film of matrix material (Figure 1b) [28,29]. Due to their remarkable energy absorption capabilities with high specific stiffness and high damping capacity [30,31,32], these foams are commonly used in foam-filled tubes, blast resistance, sound and noise insulation, foam core sandwich, shock absorbers, etc. [33]. Owing to their structure, closed-cell foams usually have a higher compressive strength and are denser, requiring more materials to manufacture. Since they are denser, they thus exhibit higher strength and need a special gas (inert gases) to fill the pores for better insulation and low thermal conductivity [34,35,36]. 

## 3. Microstructure of Metal Foams

**Figure 2:**
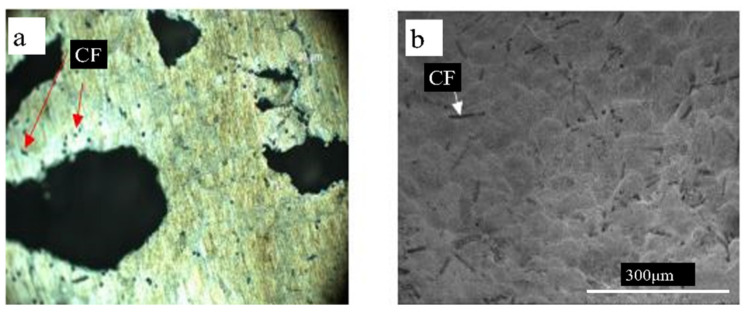
(**a**) Optical image; (**b**) SEM image of carbon fibres (CF) reinforced AlSi7 foams [37].

The microstructure of the cell-wall matrix influences mechanical properties and necessitates a controlled pore structure in metal foams. The microstructure analysis of Ni-coated carbon fibres reinforced AlSi7 foams was carried out. These foams were fabricated by a powder metallurgy technique using TiH_2_ as foaming agent. The resultant foams exhibited a uniform distribution of coated carbon fibre on the cell walls of aluminium foam, which means good wettability at the carbon fibre–Al foam interface and thus strong interfacial bonding of carbon fibres with the Al matrix was achieved. Figure 2 shows clear evidence of dispersed carbon fibres separately, stretched and aligned randomly in the aluminium matrix [37]. It was found that the stability and the maximum foam expansion for AlSi7 alloy foams depends on the size and volume fraction of SiC particles added. Increasing the particle size and/or decreasing the ceramic particle content results in a uniform cell structure formation. This was due to the cell wall thinning rate and viscosity of the Al melt affected by ceramic inclusion [38]. The A359 foams reinforced with Al_2_O_3_ particles were developed via melt foaming. These foams exhibited a closed cell, uniform cell size and roughly equiaxed polyhedral structure. In addition, the uniform distribution of the Al_2_O_3_ particles in the cell wall resulted in good interfacial interaction between the A359 matrix and Al_2_O_3_ particle [39].

**Figure 3:**
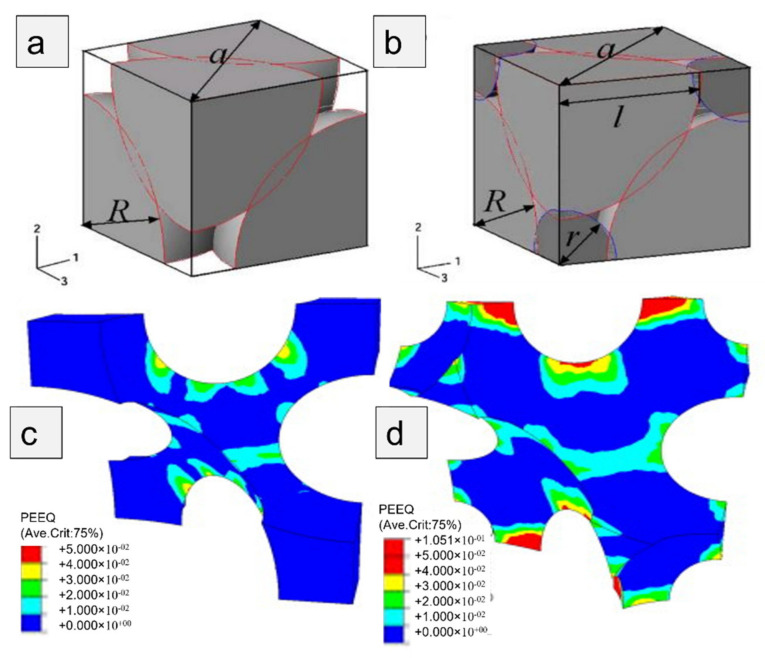
Compacted structures of fillers in (**a**) uniform-size cellular structure; (**b**) dual-size structure; Equivalent plastic strain distributions in: (**c**) uniform-size cellular structure; (**d**) a dual-size cellular structure (bimodal cell size distribution) (relative density = 0.1 and the compression strain = 2%) [40].

**Figure 4:**
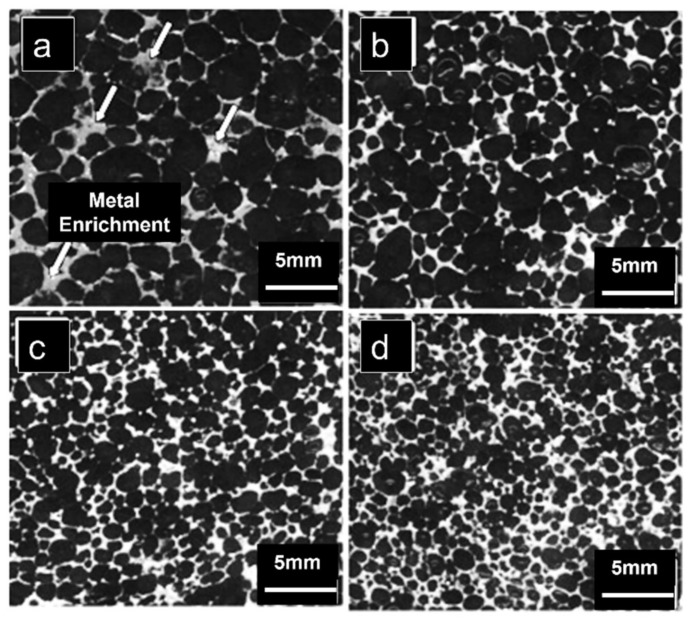
Microstructure of Mg alloy foams with varying cell sizes: (**a**) D = 1.6 mm; (**b**) D = 1.2 mm; (**c**) D = 1 mm; (**d**) D = 0.9 mm [41].

The uniform cellular structure and dual-size cellular structure (uniform-cell distribution embedded with secondary-size cells or/and bimodal cell size distribution) under compression as shown in Figure 3 were studied. The plastic deformation in the former case was concentrated in the primary inclining struts, while in the latter one, deformation was shared by both major as well as minor struts. Thus, secondary-size cells enhanced the strength and stiffness of the Al foams [40]. The pore structure of Mg alloy foams with varying cell shapes developed using melt foaming technique was analysed. The composites with porosities lower and higher than 70% exhibit spherical and polyhedron-shaped cells, respectively. In this case, due to 87% porosity, the cell shapes were polyhedron, as evident from Figure 4 [41].

**Figure 5:**
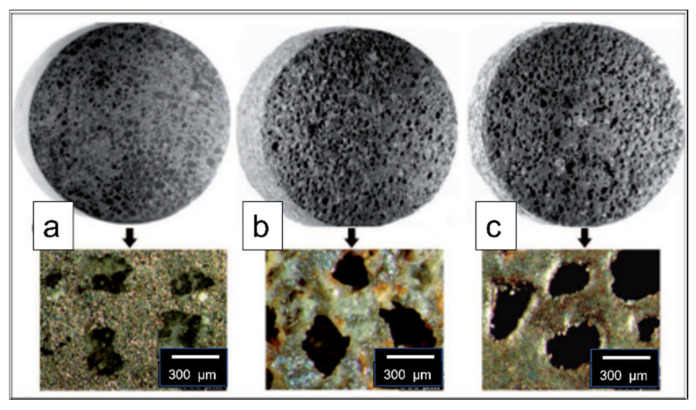
(**a**) Compacted; (**b**) Leached; (**c**) Sintered Fe(Al) foams [43].

Using potassium carbonate particles as space holders, copper foams were developed by the powder metallurgy technique. The two types of pore structure were formed: macro-pores and micro-pores. Macro-pores were interconnected spherical-shaped pores with the same size as potassium carbonate particles, while micro-pores were found in the pore struts and walls. The open-pore structure and connectivity of the macro-pores are formed by these micro-pores, resulting in foams of a high degree of pore connectivity [42]. The homogenous distribution of interconnected pores in Fe (Al) foams was obtained in a compacted specimen, as shown in Figure 5a. These pores after the leaching process replicate the size and shape of space holders (NaCl particles), as shown in Figure 5b. This demonstrates that the final morphology of pores can be tailored by selecting different types of space holders with varying sizes. Furthermore, the use of polyester resin as a binder provided adequate strength to prevent compact deformation and collapse and also reduces the corrosion and dissolution of the base metal during the leaching process. Figure 5c depicts the pore structure after sintering, when resin is removed [43]. 

**Figure 6:**
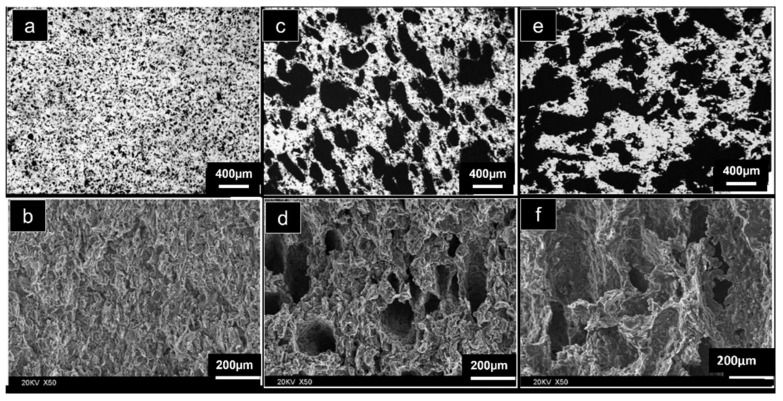
Optical and SEM images of the Mg alloy foam with different porosities: (**a**,**b**) 7%; (**c**,**d**) 36%; (**e**,**f**) 55% [46].

The selection of a proper compaction pressure is critical for producing high-strength foams because it affects their pore morphology. The influence of compaction pressure on pore morphology and densification was analysed using acrawax as space holders, which are lubricating and compressible. At lower compaction pressure (200 MPa), spherical pores were obtained, however, the cell walls were porous (micro-pores). Denser cell walls and mostly spherical pores were obtained when compacted at 300 MPa. At higher compaction pressure (400 MPa), the cell walls were more dense, but the high pressure deformed the pore morphology into an ellipsoidal shape [44]. The study of the pore microstructure of Mg/CNT composite foams developed via the powder metallurgy technique using carbamide particles as space holders revealed that the local porosity fluctuation increases as the overall porosity increases. The absolute fluctuation amplitudes are 1%, 2% and 3% for overall porosity of 29%, 39% and 49%, respectively. These variations are minor in comparison to the overall porosity and pores that are uniformly dispersed in each composite foam. Composite foams with higher overall porosity have large and many more connected pores than those with lower overall porosity. Thus, the maximum pore size as well as the total specific surface area increases significantly with the increase in the overall porosity [45]. Figure 6 depicts the optical and SEM images of these foams with varying porosities [46,47].

The effect of anodization treatment on the morphology of primary α-Al grains, as well as the constitution and distribution of secondary phases induced by adding thickening and foaming agents to Al foams developed by melt foaming, were investigated. The results showed the formation of dendritic α-Al grains and CaAl_4_ phase. The cooling curve of Al foams showed that during solidification, primary α-Al formed first, followed by CaAl_4_; thus, CaAl_4_ was not present in the melt and does not contribute to thickening [48]. In addition, the microstructure of metal foams is also influenced by the fabrication technique used. Table 1 shows the various studies on the morphology and microstructures of metal foams developed by melt foaming and powder metallurgy The microstructures of metal foams formed using different foaming agents and space holders have been mentioned [48,49,50,51,52,53,54,55,56,57,58].

**Table 1:** Microstructures of metal foams fabricated by melt foaming and powder metallurgy technique.

Manufacturing Techniques	Material	Foaming Agent/Space Holders	Microstructure	Reference
Melt foaming	Al matrix, graphene	NaCl, KCl and PMMA	- Closed pores due to incomplete dissolution of NaCl primarily due to hinderance offered by the high dense samples. - Good interfacial bonding strength between the Al matrix and the NaCl interface. - Lesser micro-porosities due to high dense material manufactured through the MI process.	[49]
	Al-Si13-MgX (X = 2.5–15 wt %) alloy	Mg	- Porous structure exhibited microporosity, broken/missing/cell walls and elliptical cells, as a result of merged pores.	[50]
	AlMg50, Ca	TiH_2_	- Uniformly distributed Mg in the matrix. - Due to the restriction effect of cell walls, the grain morphology of primary α-Al in cell walls of Al foams is irregular. - Cell-wall grains are much smaller than those in the pore-free layer.	[48]
	A356 foams	CaCO_3_	- The stabilization was achieved due to foaming gas (CO)/melt reaction during foaming producing CaO, Al_2_O_3_ and MgO. - The porosity increased with holding time. - The cell size increased with increase in CaCO_3_ content.	[51]
**Powder metallurgy**				
Using foaming agent	AlSi10 alloy	TiH_2_	- Alloy and the reinforcements are bonded metallurgically strong. - As the temperature rises to 150 C, the matrix softens and undergoes plastic deformation of the cell walls.	[52]
	Mg, Al, Cu, and Zn, yttrium	TiH_2_	- Large number of closely packed gas-filled pores. - Uniformly distributed and few elliptical pores.	[53]
	AlMg4Si8 alloy and multi-walled carbon nanotubes (MWCNT)	TiH_2_	- Good dispersion of MWCNT in aluminium alloy foam.	[54]
Space holder technique	Ti-based Cu alloy	Acrawax	- Cells obtained in the foams were nearly circular and mostly interconnected.	[55]
	Steel (iron, graphite phosphorous)	Urea granules	- Uniformly distributed spherical cells between the cell walls. - Sintering temperature and applied pressure have the weakest and strongest effect on the porosity.	[56]
	Aluminium, Graphene	NaCl, KCl, and PMMA	- Primarily composed of closed macro-pore structures. - Pore morphology same as that of space holders. - Process produced the desired closed pore structure. - With increase in volume fraction of the space holder, the cell walls became thinner, and density decreased.	[49]
	316L austenitic stainless steel	Urea particles	- Cell size was comparable to that of space-holder particles. - Cells are mostly interconnected, open, and spherical in shape. - Cell walls are larger in size. - Thinner cell walls with microporosities as a result of higher evaporation rate of the space holder. - Strong cell wall with low microporosity.	[57]
	Al matrix and MWCNT	Urea particles	- Uniformly distributed pores formed in the foam structure with shapes similar to spherical urea granules. - Large number of pores formed across the cross-section of the foams with increase in urea content.	[58]

## 4. Mechanical Properties of Metal Foams

The mechanical properties of metal foams, particularly their compressive strength and energy absorption capacities, are dependent on their cell structure, porosities, and relative densities. Table 2 shows the mechanical properties of various metal foams with varying porosities and strain rates. The plateau stress has been found to have higher values at porosity varying from 60 to 70%, while energy absorption capacities are higher for higher porosity values [59,60,61,62]. The effect of pore morphology on the compressive strength of Al foam concluded that the pore shapes of foams had a greater influence on their mechanical properties than the pore size [63]. Furthermore, the addition of TiB_2_ particles to Al foam increases the maximum foam expansion significantly due to the solid phase lowering the minimum cell wall thickness, allowing larger expansions before cell rupture and collapse. This results into higher proof stress, yield strength and more energy absorption for a given strain as compared to pure Al foams [64,65]. 

The higher values of porosities can reduce energy absorption capacity of foams. Therefore, it is essential to develop foams with optimum porosities. As the porosity of AlCu5Mn foams increases from 45.8 to 91.2%, the corresponding energy absorption capacity decreased from 72.22 to 2.70 MJ m^−3^. The energy absorption capacity has the highest values of 72.22 MJ m^−3^ at 45.8% porosity. In addition, the compression properties of AlCu5Mn foams were found to be better than those of other Al-based foams [66]. The effect of porosity on the compressive strength of TiNi foams was investigated. The study revealed that increased porosity resulted in a continuous decline in the elastic modulus and compressive strength of foams [67]. In another study, the densification strain decreased with the increment in foam density and uniform deformation in compression and strain hardening, which were the same as the bulk material with no plateau stress [68]. The compressive properties and energy absorption behaviour of powder metallurgy fabricated Al foams reinforced with glass fibres was found to depend on the volume fraction of glass fibre and porosity fraction. In addition, the compressive strength of the composite foams was higher than that of the pure Al foam [69]. The compressive strength of Cu foams under quasi-static compressive conditions concluded that at high strain levels in the densification stage, the stress–strain curves slightly depend on the strain rate [70]. Open-celled Zn foams with porosities in the range of 74 to 92% were fabricated by the space-holder technique using spherical carbamide particles as space holders. The modulus of elasticity and compressive yield strength decreased with porosity, and they have good compatibility with the Gibson–Ashby model for cellular solids [71]. The angular carbamide particles as space holders in Al foams reduced their mechanical properties significantly; however, the desired properties were obtained by using spherical carbamide particles as space holders [72].

**Figure 7:**
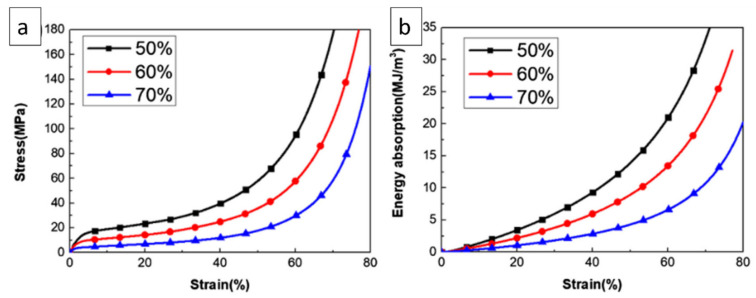
(**a**) Stress–strain curves; (**b**) Energy absorption curves of Al foams with varying porosities [60].

In an Al foam prepared by the space-holder technique, the plateau region as shown in Figure 7a exhibits a slowly progressive increase in stress, and no stress drop appears on the compression curves as a result of the hardening of foams. In addition, the uniform distribution and size of the pores also facilitates the smooth fluctuation of the stress–strain curves. The energy absorption of Al foams decreases with increasing porosity, as shown in Figure 7b. The values of Al foam parameters such as plateau stress and energy absorption at a strain rate of 60% under quasi-static compression are mentioned in Table 3 showing the same trend [60]. In addition, Table 3 shows the effect of fabrication techniques and the mechanical properties of various metallic foams developed by melt foaming and the powder metallurgy technique. The compressive strength of foams increased with the addition of reinforcements and the presence of a well-defined porous structure. This indicates the feasibility of these fabrication techniques for the development of metallic foams [65,73,74,75,76,77,78,79,80,81,82,83].

The mechanical properties of metal foam have been predicted using Gibson–Ashby mathematical models [84,85,86]. According to Gibson–Ashby, this model proposed the relationship between the relative stress and relative density and found the plateau stress or yield stress (ys) and elastic modulus E_f_ of metal foam.

σ_pl_ = σ_ys_Cρ_rel_^3/2^ (open cell) (1)

E_f_/E_s_ = ρ_rel_^2^ (open cell) (2)

**Figure 8:**
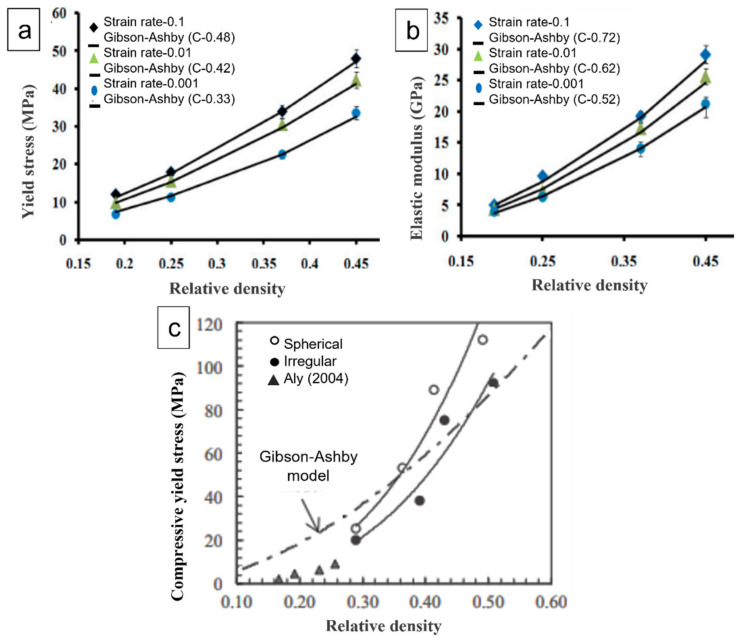
(**a**) Yield stress (σ_ys_), and (**b**) Elastic modulus (E_f_), as a function of relative density at varying stain rates [57] and (**c**) Relative density vs. compressive yield stress of Fe–1.5% Mo steel foams [88].

σ_pl_ = σ_ys_Cρ_rel_^2^ (closed cell) (3)

where σ_pl_ = plateau stress of the metal foam, σ_ys_ = yield stress of the cell metal foam; ρ_rel_ is the relative density; C = shape factor = 0.3 E_f_ = elastic modulus of the metal foam, and E_s_ = elastic modulus of the solid metal. As shown in Figure 8a,b, Jain et al. [57] investigated the compressive behaviour of austenitic stainless steel foam (ASSF) at three different compressive strain rates at varying relative densities. The stress–strain curves revealed three regions: (a) elastic, (b) plateau, and (c) densified region. The compressive yield stress and Ef values when compared to the predictions of Gibson–Ashby model agreed with the model, as evident from Figure 8a,b. For yield stress, at strain rates of 0.001, 0.01, and 0.1 s^−1^, the shape factor values were found to be C = 0.33, 0.42, and 0.48, respectively. In addition, for Ef shape factor, values were 0.52, 0.62, and 0.72 at the strain rates of 0.001, 0.01, 0.1 s^−1^, respectively. The value of ‘C’ was found to increase with strain rate. Furthermore, Jiang et al. [87] developed Al foams with different shapes of carbamide particles. The compressive strength of specimens with spherical pores was found to be greater than that of specimens with strip-shaped pores. At low relative densities, the strength values of steel foams are lower than predicted by the Gibson–Ashby model. In this model, pore walls are assumed to be solid metal. However, large pores, broken walls, anisotropic pore structure, micropores in cell walls, and non-uniform foam density significantly affect the mechanical properties of foams. 

**Figure 9:**
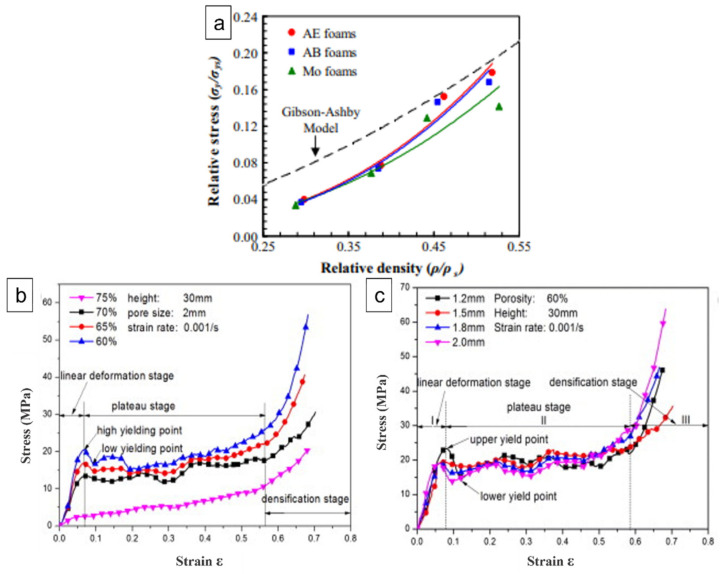
(**a**) Relationship between relative stress and relative density of the steel foams; Stress–strain curves with varying (**b**) pore sizes; (**c**) porosities [91].

Similarly, Bekoz and Oktay [88] developed steel foam using different shapes of carbamide particles. The compressive yield strengths of the steel foams obtained during the investigation were compared with the predictions of the Gibson and Ashby model in Figure 8c. The compressive yield stress at lower relative densities was lower than the values predicted by the Gibson–Ashby model. The contribution of cell face stretching to the overall strength and stiffness of foam was discovered to vary linearly with relative density. In contrast, the contribution of cell edge bending was nonlinear. The findings of Aly’s study [89] are also included in Figure 8c for comparison. It is clear from Figure 8c that the compressive yield stress is affected by relative density, and better results were obtained for foams with spherical pores. This is due to the fact that these foams exhibited comparatively smoother cell wall surfaces. However, stress concentration occurs easily at the sharp edges of irregular pores, resulting in reduced strength. According to Gibson and Ashby, the plateau stress (collapse stress) and post-collapse behaviour also depend on the type of foam, whether it is open or closed cell. Bekoz and Oktay [90] also obtained lower values of compressive yield strengths for Cu–Ni–Mo steel foams as compared to values predicted from Gibson–Ashby mathematical models, as evident from Figure 9a. It is clear that the relative stress is influenced by the relative density. In addition, the compressive yield stresses for all foams were lower than the values predicted by model, especially at low relative densities. Gibson and Ashby [86] used a simple model to analyse the yield strength of a porous metal and found that the collapse stress is not influenced by pore size [91]. The quasi-static compressive stress–strain curves of AZ31 magnesium alloy foams with different pore sizes revealed that the yield strength of specimens with pore sizes of 1.5, 1.8, and 2.0 mm were almost the same as shown in Figure 9b,c, showing consistency with Gibson and Ashby’s results [86,91], while with porosities, it varied. 

**Figure 10:**
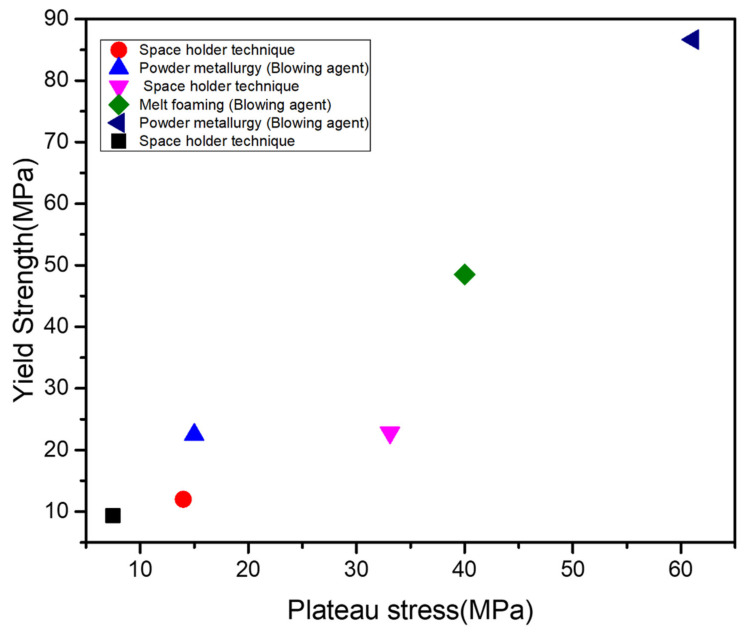
Plateau stress versus yield strength of the metal foam (Al) at a relative density of 0.4.

As it is clear that the plateau stress and yield strength are a function of relative density [86], Figure 10 shows their values by employing different fabrication techniques at same relative density of 0.4. In addition, Table 4 mentions the foam type, space holder or blowing agent used in the fabrication of Al foams. From Figure 10, it can be seen that the powder metallurgy results in increased values as compared to melt foaming. 

## 5. Fabrication Techniques of Metal Foams

Metal foams are primarily manufactured using either liquid-phase (or melt foaming) or solid-phase (powder metallurgy) techniques.

### 5.1. Melt Foaming

**Figure 11:**
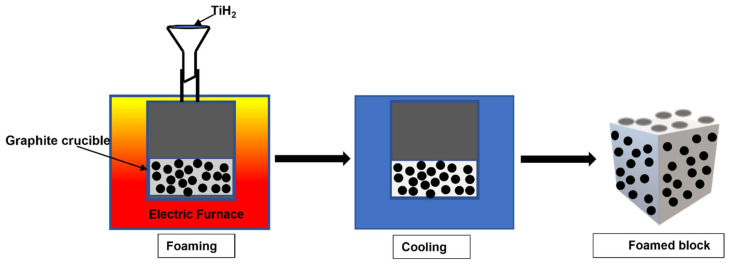
The schematic illustration of melt foaming [113].

Melt foaming is particularly popular because it produces relatively inexpensive foams with desirable properties [96,97]. The foam quality depends on various parameters, such as composition, the temperature of the forming process, holding time and cooling conditions, size, distribution and the volume fraction of reinforced foam-stabilizing particles (e.g., SiC, Al_2_O_3_, etc.) [98,99]. In melt foaming, the composite powder mix is placed in a graphite crucible and is melted using an electric resistance furnace, as demonstrated in Figure 11. The melt is maintained at a relatively low temperature to keep it sufficiently viscous when adding foaming agents or injecting gas [100].

Some of the common foaming agents and injection gases include TiH_2_ [101,102,103,104,105], CaCO_3_ [106,107], zirconium hydride [108,109], dolomite (CaMg(CO_3_)_2_) [110,111], etc. The stabilising particles such as Ca, ZrB_2_, CaO, Al_2_O_3_, etc., are also added into the melt [83,112]. The melt is then stirred continuously for the foaming agent and stabilizing particles to distribute evenly. The crucible is kept in the furnace to sustain the required temperature for decomposing TiH_2_ and releasing gas and bubbles. The foamed melt is taken out of the furnace and cooled in the air. This technique was used to fabricate A356/20SiC composite foams with varying porosities and cell sizes with TiH_2_ as a blowing agent [113].

In another study, AlSi9Mg/SiC composite foams were successfully developed using direct melt foaming with CaCO_3_ as a blowing agent and SiC as a foam stabiliser. The yield stress and collapse plateau stress of composite foams increased along with the SiC volume fraction [114]. Similarly, SiC-reinforced AlSi9Mg composite foams fabricated by this technique resulted in an increased elastic limit of composite foams along with the strain hardening [115]. In addition, Mg alloy foam was fabricated with this method to examine the microstructure of the foams [116]. Additionally, the AlSiCu cellular foams with TiH_2_ were developed to investigate the effect of the thermal decomposition of TiH_2_ on the foaming behaviour of the Al alloy. The effectiveness of the melt foaming of the Al alloy is highly dependent on the decomposition properties of TiH_2_ [117]. Mg alloys foams were fabricated by the melt processing method using CaCO_3_, TiH_2_, or MgH_2_ powders as blowing agents, which release gas during decomposition and gradually foam the magnesium alloy melt [118,119,120]. Various additives, such as SiC, carbon, and calcium particles were added to the melt to increase its viscosity [121]. Several metallic foams have been developed by using the melt foaming technique, as mentioned in Table 5, where TiH_2_ and CaCO_3_ were mostly used as foaming agents for the development of a foam structure.

### 5.2. Powder Metallurgy Foaming Techniques/Methods

In this technique, the metallic powder was mixed with some mass fraction of foaming agent (TiH_2_ powder) or space holders in a powder mixer. The mixed powders are then compacted cold, using a uniaxial compaction, in a lubricated tool-steel die at required pressures to achieve precursor green densities. The precursor specimens are later led to a furnace, so that the foaming procedure take place under high temperatures in case of green compacts with foaming agents, while for compacts with space holders, porosities are acquired by a leaching process followed by sintering.

#### 5.2.1. Using Foaming Agent

**Figure 12:**
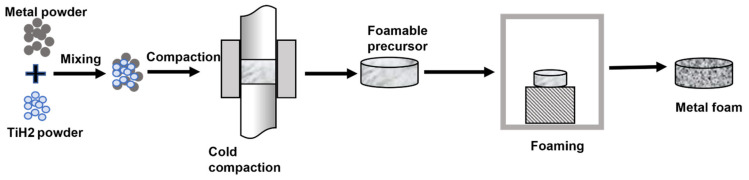
Schematic diagram of powder metallurgy technique for metallic foams.

Metallic powders and a foaming agent such as titanium hydride powder are mixed and then cold compacted, as illustrated in Figure 12. This compacted sample is then placed inside the furnace for sintering where titanium hydride decomposes, resulting in high-pressure voids. These expand by semi-solid flow, and the metal swells, forming foam that fills the mould before cooling and stabilising it. The process produces components that have a similar shape as the mould but have a lower relative density. Metals such as tin, brass, zinc, lead and bronze can also be foamed using the correct process parameters and foaming agents. The foaming agents (TiH_2_ or MgH_2_) were used to fabricate ZnAl4Cu1-alloy and AlSi12 alloy foams [131]. It was revealed that in order to foam zinc, the foam must be significantly overheated above the melting temperature of the metal, or a greater amount of foaming agent must be used. The deformation behaviour of zinc foams was comparable to that of Al foams. The compression strength of zinc foams was significantly lower at the same density but similar at equal porosity [131].

#### 5.2.2. Space-Holder Technique

**Figure 13:**
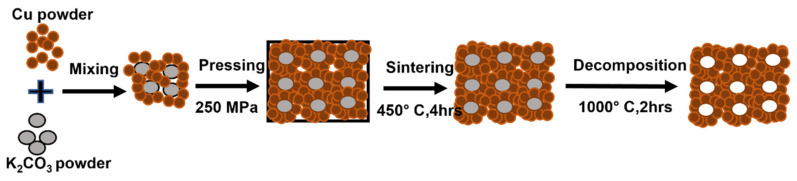
Schematic representation of the Cu foam fabricated by the space-holder technique [69].

The space-holder technique enables producing metal foams with controlled pore morphology [132]. Figure 13 shows the processing of metal foams via the space-holder technique. The space holders are firstly mixed with the metal powders. These space holders can be polymeric materials, or salts, such as NaCl particles [133,134,135,136,137,138], K_2_CO_3_ [69], carbamide particles [60,139], carbohydrate particles [140], polymethylmethacrylate (PMMA), etc. [141]. Approximately 1 to 2% binders are added to increase the strength of the final part. The mixture is compressed, or injection moulded, which is followed by sintering where space holders are removed due to heating, solvent debinding (before sintering), or by carrying out a dissolution process after sintering [142,143]. The removal of the space-holder material produces connected pores that may be open or closed. Finally, the green sample is sintered to enhance its structural strength. Overall, the space-holder method is a comparatively simple method for developing metal foams [144]. 

Many authors used this technique to produce metal foams focusing on processing parameters, feasibility, and efficiency [145,146,147]. Iron-based foams are quite often manufactured using this technique, as it allows the production of pure materials almost free from inclusions and impurities. It also enables the preparation of materials in a near-final shape with an interconnected pore structure and the required properties [148,149]. Metal foams fabricated by a pherical space holder generally show a higher compressive strength [87,150]. The effects of shape and space holder particle content were investigated; the cell wall of spherical pores was more uniform due to the increased contact between the metal particles during sintering [88]. The compressive strength of Al foams fabricated by this technique decreased with the increase in particle size of the space holder (using two particle sizes: 5–10 mm and 10–15 mm) due to the formation of thin walls. However, their energy-absorption capacity increased with the increase in the particle size of space holders, except for compacted samples [151]. Carbamide particles as space holders were employed to develop Al foams and porosities were efficiently controlled, resulting in a higher compressive strength that further increased along with sintering time and temperature [72]. Mg alloy foams were also developed using carbamide particles as space holders. The water-soluble polymers such as PMMA particles can be considered as promising space holders for developing foams. Al and Mg foams were developed through the powder metallurgy technique, using (PMMA) particles as space holders, resulting in efficient control over porosities and densities by varying the PMMA particle content. PMMA particles leave almost negligible residue on decomposition during sintering [152,153,154]. This technique has been employed to fabricate various metal foams, as mentioned in Table 6. Different foaming agents or space holders have been used to fabricate these metallic foams. 

In addition, the advantages and disadvantages of these techniques are mentioned in Table 7.

## 6. Applications of Metal Foams

Metal foams encompass a wide range of applications as mentioned in Table 8, and new uses emerge all the time. A European distributor of Alporas foams (Gleich) from Shinko developed a vacuum lifting tool for a large-scale producer (Pilkington) of flat glass products using the float glass process. Replacing the entire Al part of the tool with Alporas foam reduced the weight from 82 to 32 kg. Thus, this tool becomes handy when they are changed manually. Although the foam tools are manufactured on a small scale, i.e., five to six pieces per year, these Al foams could withstand temperatures as high as 400 °C due to their higher heat resistance. Their outstanding machinability further encourages the usage of this material [167]. 

**Figure 14:**
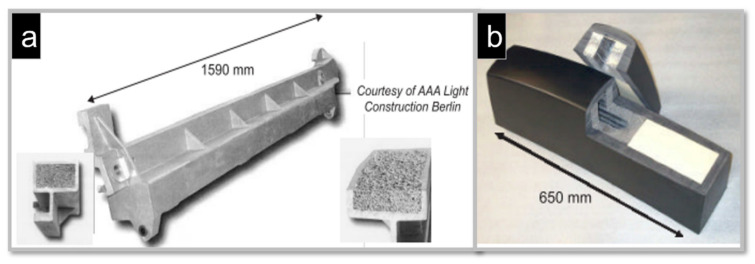
Applications of closed-cell Alporas: (**a**) a transverse beam of a machine and two insets showing its cross-sections in different directions (courtesy of AAA Light Construction Berlin); (**b**) an energy absorber for a tram built for the COMBINO vehicle system (courtesy of Hubner, Schunk, Siemens).

Another closed-cell Alporas (Al foam core) was fabricated into a composite beam (Figure 14a) by embedding the AlZn10Si8Mg alloy foam completely in the denser skin of AlZn10Si8Mg alloy using the sand-casting technique. The transverse beam dampened vibration frequencies up to 370 Hz by internal friction or interface slipping between the core and the skin. This part was equipped in 700 machines approximately so far. In this frequency range, sound attenuation of up to 60% was achieved [181]. Since 2004, the company (Gleich) has been producing 2500 parts, each weighing 21 kg. Figure 14b shows the system of the modular tram concept (COMBINO) produced by three German manufacturing companies: Siemens (tram), Huebner (impact absorber), and Schunk Sintermetalltechnik (metal foam). This system meets customer needs basing on the same framework. In addition, the Al foam core for the impact energy absorber was developed by the extrusion of powder mixtures, which was followed by the embedment of a rubber shell with the foam. These absorbers are manufactured in hundreds of units for other tram operators and manufacturers [182].

**Figure 15:**
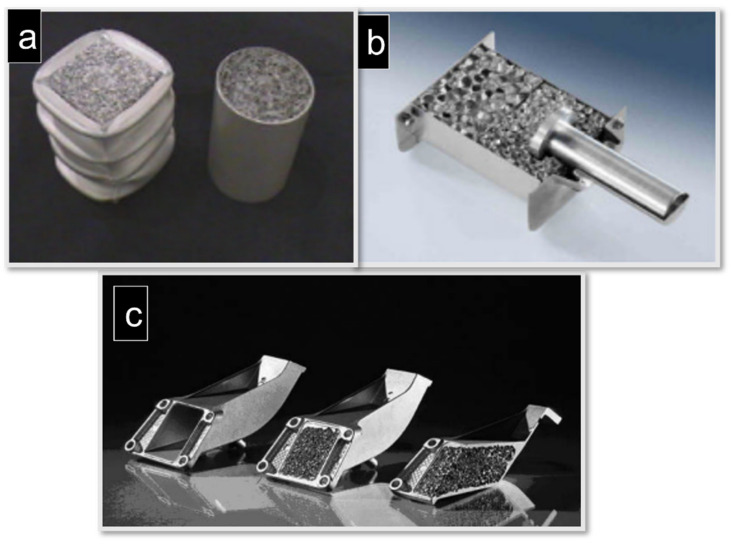
Applications of metal foams: (**a**) prototypes of crash absorbers made of extruded Al filled with Cymat foam core (courtesy of Cymat); (**b**) design based on Al foams (Metcomb) with two different densities (courtesy of Hutte Kleinreichenbech); (**c**) prototype of a BMW engine mounting bracket produced by LKR Ranshofen. From left: empty casting, composite part comprising foam core and cast shell, and section through composite part (courtesy of LKR).

Figure 15a shows a crash box for the front-end module systems of Valeo, which was designed by Cymat and Valeo in a joint development programme. Figure 15b shows a slightly different design concept, consisting of foams with varying densities for adjusting the absorber’s deformation curve [181]. The Austrian manufacturer Alulight manufactures 100,000 pieces of closed-cell Al foams annually as a crash absorber element for Audi cars, making it the only large-scale production of closed cell-Al foams through automated production technology [183]. The automobile manufacturer BMW (München, Germany) and LKR (Ranshofen, Austria) jointly developed an engine mounting bracket using Alulight foams as a lightweight inner core (Figure 15c). This bracket could bear the heavier weight of the car engine and mechanical vibrations by dissipating thermal energy. The high fracture toughness and stiffness of these composites were improved, resulting in enhanced safety in crash situations [181]. 

Alcoa (the USA) developed a new method based on the continuous casting technique to reduce the cost of Al foams for inexpensive products [184]. The technology has matured to mass produce low-cost foams. AlSi alloy were used as a filling foam in the crash element, with a 30% improvement in absorbing the impact energy, while the vehicle weight increased by 3% only [185]. NiMH and NiCd battery electrodes are nowadays perhaps the primary industrial application for metal foams. Vale Inco manufactures 4 million m^2^ of Ni foam annually for these applications [186]. Ti-based foam with ammonium bicarbonate (NH_4_HCO_3_) as a space holder was developed by powder metallurgy for bone implant application [187].

## 7. Challenges and Way Forward

The technology of metal foaming is developing at an accelerated pace with considerable progress. This research area involves interdisciplinary collaborations among physics, chemistry, and materials engineering to produce the required quality economically with reproducible foamed materials that possessed a unique range of properties. These diverse applications allow the construction of functionally new parts or devices. Since foams have many competitors that are mostly less expensive, it becomes crucial to improve fabrication technologies for large-scale foam production, generating a greater variety of low-cost metal foam products. Despite the successful development, more basic research remains essential to understanding the correlation between the composition of metal alloy and foamability. Producing foams of consistent quality, morphology, and control of structure is challenging. However, the foam quality and properties can be controlled by optimizing the factors and parameters. In addition, the foam’s porosities can be controlled by heat treatment or coating the blowing agent to stop the decomposition of the gas-blowing agent in the melt. Overall, to produce uniformly structured metal foams, it is necessary to optimize the processing parameters in the existing fabrication techniques. In addition, further studies are required to compare the available processes to produce metal foams from raw material (powders) or Al scrap. The development of alloy foams for the core with improved properties may contribute to their successful usage in various applications.

The academic and industrial research plays a crucial role in eradicating problems that otherwise limit the wider applications of metal foams. Improvement is required to ensure the reproducibility and uniformity of the foam’s cell structures for attaining even densities and distribution of pore sizes in the entire part or component, resulting in uniform foam structures. Additionally, new processing routes, cheaper raw materials, and less wastage may reduce the processing and material costs of metal foams. Case studies based on simulation, innovative design, and testing could show end-users that despite the higher costs of some foams or parts, there are more benefits associated with these new structures and materials, such as savings in weight and energy.

## 8. Summary

This review presents an overview of the metal foams developed by various techniques to enhance their properties and performance, summarising the efforts to improve the foaming behaviour and characteristics. Depending on their applications, foam structures could be tailored to generate open- or closed-cell foams. Microstructural studies evaluated the effect of reinforcements and fabrication techniques on the foams’ microstructure, and it defines their mechanical behaviour. The foams’ properties, particularly the compressive strengths and energy absorption, could be improved by adding different reinforcements, such as metal or ceramic particles, to stabilise the foam structure. The porosities in these foams are dependent on foaming agents and space-holder particles. The processing techniques that fabricate metal foams play a vital role in deciding their properties and foaming behaviour. Their applications as structural components in automotive parts, such as ships and aerospace transportation, and as sound dampers, filters, electrodes, etc. can be effectively attained. However, improvement would be possible by identifying the downsides and feasible solutions for better performances under various working conditions and production techniques. Although metal foams find applications in many sectors due to their lightweight and high strength, the high cost of fabrication techniques hinders large-scale production. The challenge of future process development should therefore focus on reducing the production cost as well.

## Figures and Tables

**Table 2:** Mechanical properties of metal foams with varying porosities and strain rates.

Fabrication Technique (Strain Rate)	Material	Porosity	Plateau Stress (MPa)	Energy Absorption (MJ/m^3^)	Reference
Space-holder technique (Strain rate = 0.01/s)	Ti foam	80	12.55	-	[59]
	Ti foam	78	15.42	-	
	Ti foam	76	15.84	-	
	Ti foam	74	21.61	-	
	Ti foam	72.4	25.43	-	
	Ti foam	70	27.97	-	
	Ti foam	66.6	30.76	-	
Space-holder technique (Strain rate = 0.01/s)	Al foam	50	29.5	20.9	[60]
	Al foam	60	18.8	13.5	
	Al foam	70	9.9	6.6	
Melt foaming (Strain rate = 3 × 10^−3^ s^−1^)	Al/0.25 wt.% SiO_2_	86	0.8	13.7	[61]
	Al/0.5 wt.% SiO_2_	84	1.4	46.2	
	Al/0.75 wt.% SiO_2_	91	0.4	23.0	
	Al/1.0 wt.% SiO_2_	87	0.7	18.3	
Space-holder technique (Strain rate = 0.01/s)	Ti foam	68	100	120	[62]
	Ti foam	57	180	160	
	Ti foam	46	260	220	

**Table 3:** Mechanical properties of various metallic foams developed by melt foaming and powder metallurgy technique.

Foam Material	Foaming Agent	Fabrication Technique	Mechanical Properties	References
Al (ALPORAS)	TiH_2_	Melt foaming	- Energy-absorption capacity and the plateau stress were not dependant on the distribution of cell size	[73]
Al alloy	TiH_2_	Melt foaming	- Zn and Mg strengthened Al - Compressive strength of the foams were twice as high as that of conventional foams (ALPORAS)	[74]
Mg–Al, Mg–Zn and Mg–Cu foams	CaCO_3_	Powder metallurgy	- Adding alloying metal to Mg led to efficient foaming by forming low melting temperature intermetallic compounds during sintering	[76]
Al/ scandium	TiH_2_	Melt foaming	- Adding scandium improved the compressive strength	[77]
Al/TiB_2_	TiH_2_	Powder metallurgy	- TiB_2_ particles enhanced expansion in the foam without affecting foam stabilisation - Composite foams possessed higher proof stresses and absorbed more energy	[65]
Zn foam	TiH_2_	Powder metallurgy	- Zinc oxide stabilised the foams - With increase in the oxide content, the maximum expansion and expansion rate increased	[78]
Al/3.7% Si/0.18% Mg	TiH_2_	Melt foaming	- Porosity decreased but a uniform pore structure was obtained by increasing the foaming temperature - Foams were stabilised at high temperatures (without using oxide particles or metal calcium granules) - Energy absorbed per unit mass was improved - Smooth plateau stress region due to uniform cell walls and pore morphology	[79]
Al_63_Cu_28_Fe_9_ alloy	_-_	Melt foaming	- Plateau stress and maximum stress of 30 and 80 MPa, respectively, were achieved	[80]
Al/Al_2_O_3_	_-_	Powder metallurgy	- Al_2_O_3_ addition improved the compaction and hardness properties	[81]
Zn–Mg alloy foam	CaCO_3_	Powder metallurgy	- Foam exhibited good mechanical strength but has a serrated compressive stress–strain curve during the plateau region due to intermetallics	[82]
Al/Zn foams	CaCO_3_	Melt foaming	- Increases in cell wall thickness and melt viscosity due to the formation of oxide phases in the melt - Foam with 4 wt % Zn had uniform cell structure and thus exhibited a longer plateau region and high yield strength - Energy absorbed per unit volume and foam density increases with increase in Zn content	[83]
Mg/Al/Zn foams	CaCO_3_	Powder metallurgy	- Higher compressive strength was acquired for foams as compared to other Mg or Mg alloy foams	[75]

**Table 4:** Information regarding Figure 10.

Processing Technique	Foam Type	Material	Space Holder/Blowing Agent	Reference
Space-holder technique	Closed cell	Al	Space holder (Carbamide)	[92]
Space-holder technique	Open cell	Al-Al_2_O_3_	Space holder (Carbamide)	[93]
Powder metallurgy (blowing agent)	Closed cell	Al	Blowing agent (Dolomite)	[11]
Space-holder technique	Closed cell	Al-CNT	Space holder (Carbamide)	[94]
Melt foaming	Closed cell	AlMnCu	Blowing agent (TiH_2_)	[66]
Powder metallurgy (blowing agent)	Closed cell	AA7075/SiC	Foaming agent (CaCO_3_)	[95]

**Table 5:** Metal foams developed by melt foaming.

Foam Material	Foam Type	Foaming Agents	Reference
Al	Closed cell	TiH_2_	[122]
Al/SiC	Closed cell	TiH_2_	[123]
Al/Ca	Closed cell	TiH_2_	[124]
Al 6061/Cu	Closed cell	TiH_2_	[125]
ZA22/SiC	Closed cell	CaCO_3_	[126]
Zn/22Al/SiC	Closed cell	CaCO_3_	[127]
Al alloy (ALPORAS)	Closed cell	TiH_2_	[128]
Al (ALPORAS)	Closed cell	TiH_2_	[129]
Al/Si/Mg	Closed cell	CaCO_3_	[130]

**Table 6:** Metal foams fabricated by powder metallurgy technique.

Foam Material	Foaming Agent/Space Holders	Fabrication Technique	Reference
Cu	Potassium carbonate	Space-holder method	[155]
Cu	Potassium carbonate	Space-holder method	[156]
Cu/CuO	Oxide	Powder metallurgy	[157]
AlSi10Mg	TiH_2_	Powder metallurgy	[158]
Al/Mg	NaCl	Space-holder method	[159]
Al/Y_2_O_3_	NaCl	Space-holder method	[133]
Al/SiC	TiH_2_	Powder metallurgy	[28]
Al–Sn foams	TiH_2_	Powder metallurgy	[160]
Al 6061-Al_2_O_3_	TiH_2_	Powder metallurgy	[161]
Al-Sn (Co, Mg, Mn, Ni, and Ti)	TiH_2_	Powder metallurgy	[162]
Al6061 and AlSi7 alloys	TiH_2_	Powder metallurgy	[163]
Fe/Titanium (Ti)	CO_2_	Powder metallurgy	[164]
AA2014-SiC	Calcium hydride	Liquid metallurgy	[165]
Al/MWCNTs	TiH_2_	Powder metallurgy	[166]

**Table 7:** Advantages and disadvantages of melt foaming and powder metallurgy technique.

Fabrication Techniques	Advantages	Disadvantages
Melt foaming	The melt is easily stabilised. Suited for continuous processing, with the ability to produce bulk metal foam. Economically appealing. Lighter metal such as aluminium alloys can be easily used in fabrication process due to their lower density and non-oxidising behaviour when exposed to other gases or air comprising O_2_. The process is relatively simple and straightforward.	Cannot be employed to create complex shapes and structures. Products are expensive due to high cost of metal hydride. Difficult to control cell size and porosity. Thermal stresses and cracks occur in the cell walls on removal of mould from furnace or by rapid cooling.
Powder metallurgy	Complex parts can be produced near to net shape. High-quality foams are manufactured and do not require machining/finishing. It is applicable to a wide range of metals and alloys like lead, brass, Zn, etc. Pore size and porosity can be tailored by space-holder size and quantity.	Method is expensive. Approach is not instantaneous. Fabrication process is time consuming.

**Table 8:** Various potential applications of metal foams.

Foams	Applications	Reference
Mg foams	Bone implants	[46]
Metallic foam	Heat exchanger	[168]
Ni-Cu	Electrodes for super capacitors	[169]
Al7075 and 6061 alloy	Crash boxes	[170]
	Drug delivery	[171]
Al alloy (AlSi12 or 6061)	Foaming around fastening elements	[172]
A356/steel	Radiation shielding	[173]
Al-foam (Duocel^®^)	Military-medium tactical vehicles	[174]
Fe/Mg/CNT foam	Bone implant	[175]
Al foam	Crash box for Valeo’s front-end module systems	[176]
Fe/P foam	Bone replacement	[177]
Al foam	Ship structure	[178]
Cu foam	Heat exchangers	[179]
Alulight	Tail lifts, Alimex panel	[180]
